# A Novel Role for the Transcription Factor Cwt1p as a Negative Regulator of Nitrosative Stress in *Candida albicans*

**DOI:** 10.1371/journal.pone.0043956

**Published:** 2012-08-29

**Authors:** Adnane Sellam, Faiza Tebbji, Malcolm Whiteway, André Nantel

**Affiliations:** 1 Institute for Research in Immunology and Cancer (IRIC), University of Montreal, Montréal, QC, Canada; 2 Biotechnology Research Institute, National Research Council of Canada, Montréal, QC, Canada; 3 Department of Biology, McGill University, Montréal, QC, Canada; 4 Department of Anatomy and Cell Biology, McGill University, Montréal, QC, Canada; New Jersey Medical School, University of Medicine and Dentistry of New Jersey, United States of America

## Abstract

The ability of *Candida albicans* to survive in the presence of nitrosative stress during the initial contact with the host immune system is crucial for its ability to colonize mammalian hosts. Thus, this fungus must activate robust mechanisms to neutralize and repair nitrosative-induced damage. Until now, very little was known regarding the regulatory circuits associated with reactive nitrogen species detoxification in fungi. To gain insight into the transcriptional regulatory networks controlling nitrosative stress response (NRS) in *C. albicans* a compilation of transcriptional regulator-defective mutants were screened. This led to the identification of Cwt1p as a negative regulator of NSR. By combining genome-wide location and expression analyses, we have characterized the Cwt1p regulon and demonstrated that Cwt1p is directly required for proper repression of the flavohemoglobin Yhb1p, a key NO-detoxification enzyme. Furthermore, Cwt1p operates both by activating and repressing genes of specific functions solicited upon NSR. Additionally, we used Gene Set Enrichment Analysis to reinvestigate the *C. albicans* NSR-transcriptome and demonstrate a significant similarity with the transcriptional profiles of *C. albicans* interacting with phagocytic host-cells. In summary, we have characterized a novel negative regulator of NSR and bring new insights into the transcriptional regulatory network governing fungal NSR.

## Introduction

Phagocytic cells such as macrophages and neutrophils form a part of the innate immune system and are considered as the first line of host defense against pathogens. These cells are able to ingest and subsequently kill infectious agents by producing a variety of toxic chemicals. The most important of these are antimicrobial peptides, nitric oxide (NO), the super oxide anion (O_2_^−^) and hydrogen peroxide (H_2_O_2_). In mammalian cells, NO is produced by a high-output form of nitric oxide synthase [1]. Upon phagocytosis, macrophages release reactive nitrogen species (RNS) and reactive nitrogen intermediates (RNI) into the phagolysosome to neutralize the engulfed pathogens. In addition to its direct antimicrobial role, NO reacts with the super oxide anion O_2_^−^ to create the strong oxidant peroxynitrite (ONOO^−^), which has fungicidal activity [2].

*Candida albicans* is an opportunistic pathogen responsible for various non-life-threatening infections that can become very serious in immunocompromised patients. The ability of this commensal yeast to colonize numerous sites and organs requires that it can adapt rapidly to a variety of different environmental stresses such as reactive oxygen species (ROS) and RNS generated by phagocytic cells. The protective role of RNS against *C. albicans* was established in many infection models including murine oral candidiasis [3]. The ability of *C. albicans* to survive in the presence of nitrosative stress during the initial contact with the host immune systems is important for colonization. Thus, this fungus must activate robust mechanisms to detoxify RNS and repair NO-induced damage. Previous works had highlighted the role of the flavohemoglobin enzyme Yhb1p in NO detoxification through its action in converting the short NO radical to nitrate [4]. The *C. albicans yhb1* homozygous mutant is hypersensitive to NO killing and exhibits a slightly reduced virulence in a tail vein model of disseminated candidiasis [5]. The transcription level of *YHB1* is highly induced by NO and its inducibility under nitrosative stress depends on the Zn(II)_2_-Cys_6_ transcription factor (TF) Cta4p [6], [7]. A nitric oxide-responsive cis-regulatory element recognized by Cta4p in the promoter region of the flavohemoglobin *YHB1* gene has been identified.

Compared to mechanisms controlling the detoxification of ROS in fungi, very little is known regarding the transcriptional circuits and signaling pathways associated with RNS detoxification. To gain insight into these regulatory mechanisms in *C. albicans* we screened a compilation of mutants from various publicly available libraries [8]–[12], concentrating on mutants for genes encoding TFs as well as components of chromatin remodeling and histone modification complexes. This comprehensive work led to the identification of a key TF that modulates the intensity of the nitrosative stress response (NSR) in *C. albicans*. Cwt1p was previously characterized as a regulator of cell wall damage and morphogenesis in *C. albicans* [13]. By combining genome-wide location and expression analyses, we have characterized the Cwt1p NSR-dependent regulon and demonstrate that this TF is required for proper repression of the flavohemoglobin Yhb1p which is a key NO-detoxification enzyme in *C. albicans* and other pathogenic fungi. This study also demonstrates that Cwt1p plays a role in both activating and repressing genes of specific functions solicited upon nitrosative stress response. Using Gene Set Enrichment Analysis (GSEA) we have revisited the *C. albicans* transcriptome in response to nitrosative stress and revealed a correlation with transcriptional programs of *C. albicans* interacting with host-cells, such as poly-morphonuclear neutrophils [14]. This resemblance strongly supports the hypothesis that immune cells such as neutrophils partly inhibit *C. albicans* through the release of NRS.

## Results

### Genome-wide Identification of Transcriptional Regulators Controlling Nitrosative Stress Response

**Figure 1:**
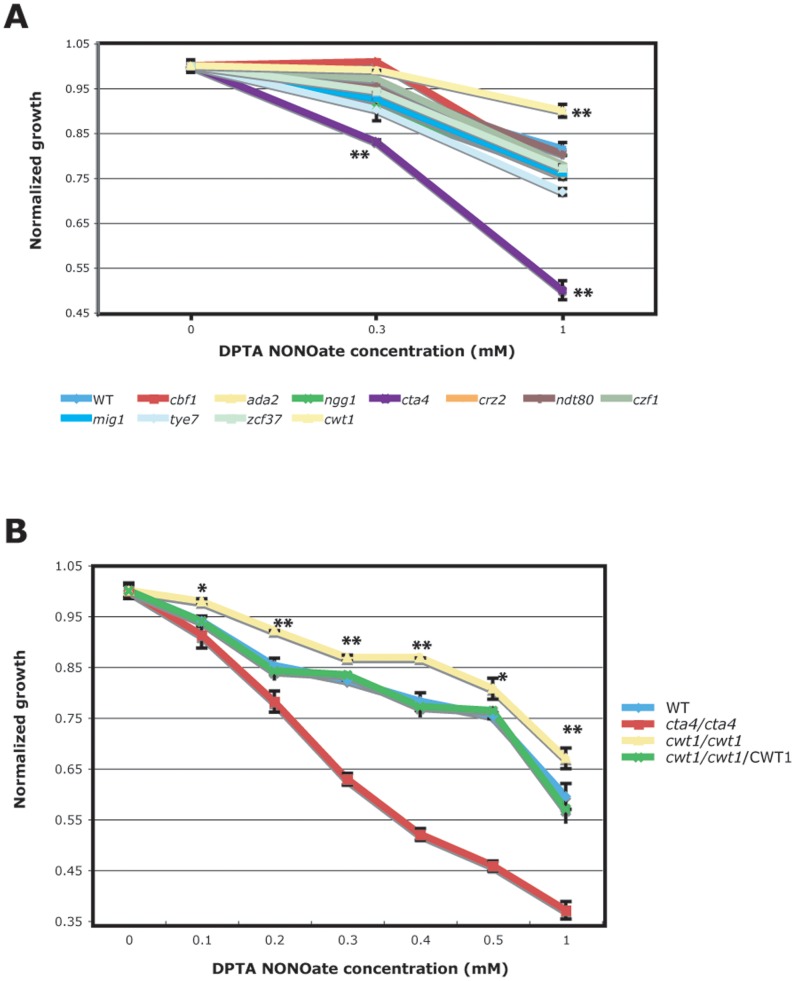
Growth inhibition assay by the nitric oxide donor DPTA NONOate. (**A**) Growth inhibition assay of selected mutant strains exposed to 0.3 and 1 mM DPTA NONOate during 3.5 hours. Growth inhibition rate was normalized relative to the 0 mM DPTA NONOate condition for each strain. Results are from three experiments. (**B**) Growth defect of *cwt1* and *cta4* strains was confirmed by an independent NO inhibition assay using different DPTA NONOate concentrations: 0, 0.1, 0.2, 0.3, 0.4, 0.5, and 1 mM. Growth of the wt (SN152) and *cwt1*/*cwt1*/CWT1 strains is also shown. Values are average of three replicates. Error bars are standard deviations of triplicates. The symbol (*) and (**) indicate a significant difference compared to the WT strain using *t*-test (P<0.0005 and P<0.0001, respectively).

We screened a compilation of 390 mutants, representing 229 transcriptional regulators, from publicly available libraries (Table S1). The ability of each mutant to exhibit sensitivity to 0.3 mM and 1 mM of DPTA NONOate (nitric oxide donor) was assessed using a liquid growth inhibition assay as described in the methods section (Figure 1A and Table S1). After retesting the observed mutant phenotypes using additional DPTA NONOate concentrations (0.1, 0.2, 0.3, 0.4, 0.5 and 1 mM), we have confirmed growth rate alterations for two TF mutant strains: *cta4* and *cwt1* (Figure 1B). As shown in Figure 1B while the *cta4* mutant strain was hypersensitive to nitrosative stress, as formerly reported [6], *cwt1* exhibited a moderate, but statistically significant increase in its resistance to NO compared to its parental strains SN152 (His+, Leu+). Since Cta4p was already characterized as a master modulator of nitrosative stress response in *C. albicans* we focused our investigation on the TF Cwt1p.

### Location Profiling of Cwt1p Genomic Occupancy

To gain a more comprehensive insight into the biological processes that are controlled by Cwt1p in *C. albicans*, we set out to determine its genomic occupancy using chromatin immunoprecipitation coupled to microarray analysis (ChIP-Chip). Binding locations were determined in duplicate ChIP-chip experiments using microarrays containing 5449 70-mer oligonucleotides representative of all intergenic regions. Statistical analysis using the Welch *t*-test with a false-discovery rate of 5% and 1.6 fold enrichment cutoff provided evidence for Cwt1p binding at 130 intergenic regions corresponding to 141 gene promoters (11 probes in our dataset represent shared promoter regions of two divergent ORFs) (Table S2 and S3).

**Figure 2:**
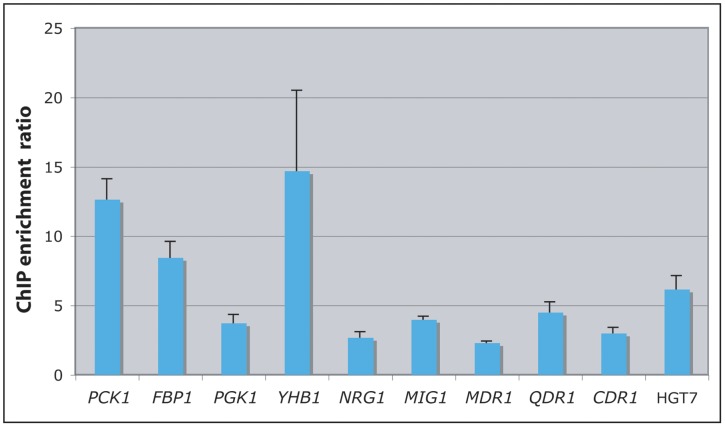
Confirmation of ChIP-chip by ChIP-Qpcr. *In vivo* occupancy of Cwt1p at ten different intergenic regions. Immunoprecipitated DNA was subjected to qPCR to validate Cwt1p binding at 100–200 bp regions surrounding the probes showing a significant binding ratio. SDs were based on data from two independent experiments.

To assess the reliability of the ChIP-Chip assays, the immunoprecipitated DNA from two additional independent ChIP experiments were quantified using qPCR. A total of 10 promoters were randomly selected and pairs of primers were designed to amplify 100–200 bp regions surrounding the probes that showed a significant Cwt1p-binding enrichment. The results obtained for the 10 selected promoters confirmed the binding of Cwt1p to the promoters identified by ChIP-Chip (Figure 2).

The target sites of Cwt1p were analyzed for the presence of potential consensus sequence binding motifs using the motif-based sequence analysis tool MEME [15]. A collection of statistically significant DNA mini-motifs was obtained and is listed in Table S4. However, none of them resemble to a typically zinc cluster consensus.

**Figure 3:**
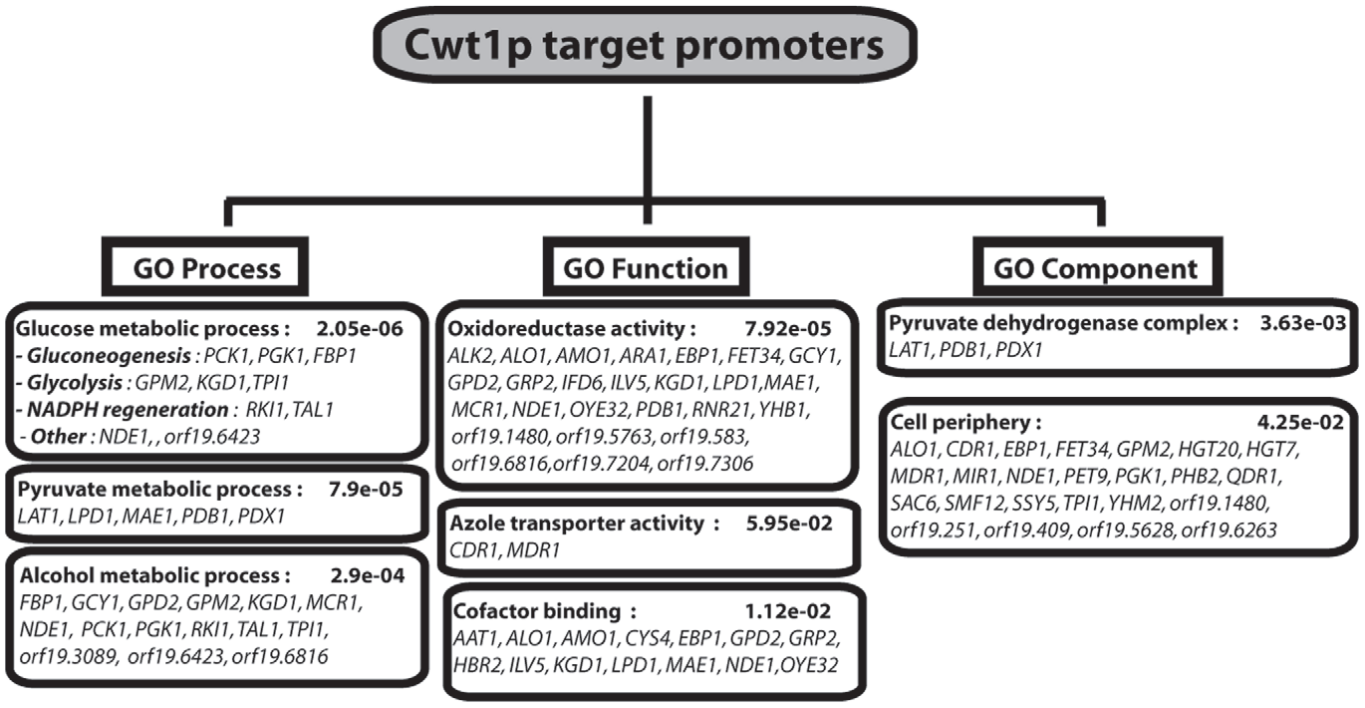
Gene ontology analysis of Cwt1p-bound promoters. GO biological process, molecular function and cellular component annotations of Cwt1p bound promoters. *P*-values were calculated using hypergeometric distribution exploiting the Generic GO Term Finder (http://go.princeton.edu/cgi-bin/GOTermFinder).

To explore the biological processes controlled by the TF Cwt1p, we conducted a gene ontology (GO) investigation by analyzing the 141 genes whose promoters are associated with Cwt1p. As shown in Figure 3, genes associated with carbohydrate metabolism including gluconeogenesis, glycolysis, pyruvate metabolisms and hexose transport were significantly enriched in our dataset (Table S3). Examination of Cwt1p target genes revealed that they were enriched in the promoters of genes coding for TFs and general transcriptional regulators including the key repressors Nrg1p and Mig1p that control genes involved in sugar metabolism**.** Cwt1p also bound the promoter regions of the azole transporters Cdr1p and Mdr1p in addition to six genes (among fifteen) that were constitutively activated in azole-resistant clinical strains overexpressing Mdr1p (orf19.7306, *MDR1*, *IFD6*, *OYE32*, orf19.7042 and orf19.7166) [16]. Cwt1p targets were notably enriched in other functional categories such as stress response, amino-acid metabolism, proteolysis, translation and the cell wall (Table S3).

### Cwt1p Binds the Promoter Region of the Nitric Oxide Dioxygenase *YHB1* and Negatively Modulates its Transcription

**Figure 4:**
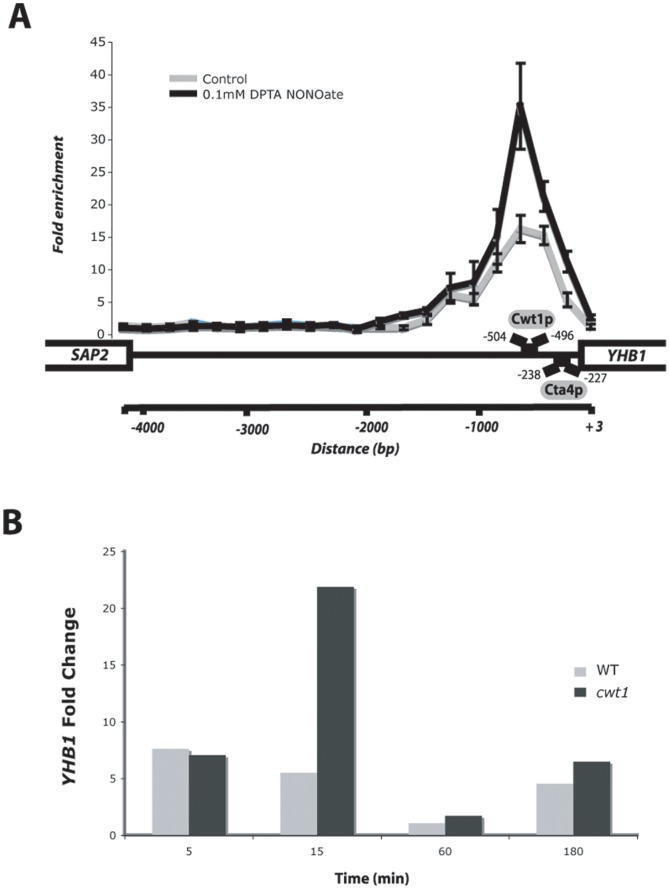
Cwt1p bound the promoter region of the nitric oxide dioxygenase *YHB1* and controls negatively its transcription. (**A**) Cwt1p is detected at the *YHB1* promoter using tiled ChIP-qPCR, and the enrichment signal overlaps precisely its DNA-binding motif. Cwt1p occupancy was assessed in the presence and the absence of nitrosative stress. The nitric oxide-responsive element (NORE) recognized by the TF Cta4p is also shown. (**B**) Average expression of the nitric oxide dioxygenase *YHB1* in response to 0.1 mM DPTA NONOate is shown in the wt and *cwt1* strains in two independent biological replicates. Fold changes were estimated by using the coding sequence of the *C. albicans* ACT1 ORF as a reference. Fold enrichments of the tested coding sequences were estimated using the comparative ΔΔCt method.

Cwt1p was found to occupy the promoter of the flavohemoglobin/nitric oxide dioxygenase Yhb1p, a key enzyme of NO detoxification. Cwt1p was also detected at the *YHB1* promoter using tiled ChIP-qPCR, and the enrichment signal overlaps precisely with the carbon source response elements (CSREs) motif (AANYCCGA) recognized by the homologue of Cwt1p in *S. cerevisiae*, Rds2p [17], [18] (Figure 4A). Cwt1p binding intensity at the *YHB1* promoter was augmented significantly when *Candida* cells were challenged with DPTA NONOate compared to the control experiment suggesting a dynamic regulatory role of this TF during NSR. In order to assess the contribution of Cwt1p to the transcriptional regulation of this key NO-detoxifying enzyme, qPCR was used to evaluate the expression level of *YHB1* in wt and *cwt1* mutants under nitrosative stress condition at different time-points (5, 15, 60 and 180 min). As anticipated, the transcript levels of the *YHB1* in wt cells were significantly induced when cells were challenged with the NO donor DPTA NONOate (Figure 4B). In the *cwt1* mutant, *YHB1* was induced similarly to the wt strain after exposure to DPTA NONOate for 5 min. However, the intensity of the NONOate transcriptional activation of *YHB1* was further enhanced in the *cwt1* mutant (Figure 4B) compared to the wt strain at 15 min, 60 min and 180 min. This demonstrates clearly that Cwt1p is a negative and direct transcriptional regulator of the NO-detoxifying enzyme *YHB1*. In the *cwt1* mutant, *YHB1* transcript level was the same as in wt after 5 min exposure to DPTA NONOate (Figure 4B). This suggests that Cwt1p-mediated repression occurs after the induction of *YHB1* and might happens to modulate *YHB1* inducibility by the TF Cta4p.

### Gene Expression Analysis of Cwt1p Target Genes

As a preliminary step to assess the contribution of the TF Cwt1p on the NSR transcriptional program, NO-responsive genes were first identified by analyzing the transcriptional profile of wt *C. albicans* exposed to DPTA NONOate for 15 min (Table S5). In response to DPTA NONOate, the wt strain induced transcription of genes involved in iron transport/assimilation (*FET34, SIT1, CTR2, FTR1, FRP6, FRP1, FTH1, HAP43*) in addition to genes related to oxidative stress response (*TTR1, ALO1, AHP1, HSP12*). Transcript levels of genes involved in cell detoxification process such as the flavohemoglobin *YHB1* and the glutaredoxin *TTR1* were also induced (Table S5). Repressed transcripts were enriched in many functions, such as response to drug (*PDR16*, *TPO3*, *FCR1*, *GCY1*, *DIP5* and *CRZ1*) and nitrogen utilization (*OPT1*, *MEP1* and *IFC3*). Gene Set Enrichment Analysis (GSEA) was used to determine whether defined lists (or sets) of genes exhibit a statistically significant bias in their distribution within a ranked gene list [19]. Expectedly, as represented in Table 1 and Figure S1, genes upregulated and downregulated in response to DPTA NONOate exhibit a significant similarity with transcripts induced and repressed, respectively, during nitrosative stress in *C. albicans* as reported previously by Hromatka et al. [7]. GSEA analysis of DPTA NONOate-induced genes further revealed a meaningful enrichment with transcripts upregulated in different *in vivo* infection models including a reconstituted human oral epithelium model (RHE) [14] and upon polymorphonuclear neutrophil-mediated phagocytosis [20] (Table 1 and S6).

**Table 1:** Summary of Gene Set Enrichment Analysis of nitrosative stress response in *C. albicans*.

Gene set	NES#
Positive correlation with the profile	
- Transcripts upregulated in response to ciclopirox [**23**]	3.69
- Transcripts upregulated in Δ*sko1* [**30**]	3.33
- Transcripts upregulated in response to nitric oxide [**4**]	3.63
- Transcripts downregulated in Δ*dfg16* [**31**]	3.74
- GO Process: Transition metal ion transport	3.27
- Transcripts upregulated upon phagocytosis by polymorphonuclear neutrophils **32]**	3.21
- GO Process: iron transport	2.49
- Transcripts downregulated in *Δcdc53* **33]**	3.93
- Tye7p-bound promoters **34]**	2.86
- Gal4p-bound promoters **34]**	3.23
- Transcripts upregulated in reconstituted human oral epithelium damaged by *C. albicans* **35]**	2.97
Negative correlation with the profile	
- Transcripts downregulated in response to nitric oxide	−3.46
- Transcripts differentially regulated during the S-G2 transition [**33**]	−3.03
- GO Process: mRNA transport	−2.56

# **Normalized Enrichment Score (NES):** a metric used to estimate statistical significance using an empirical phenotype-based permutation test procedure that preserves the complex correlation structure of the gene expression data [19]. NES adjust the Estimated Significance (ES) level to account for multiple hypothesis testing when an entire database of datasets is considered.

**Figure 5:**
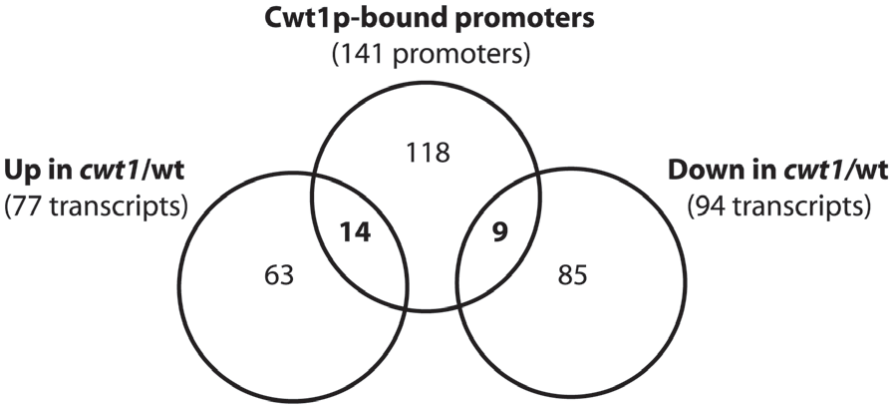
Cwt1p is a direct regulator of nitrosative stress responsive-genes. Relationship between Cwt1p-bound genes and genes showing altered expression in the *cwt1* mutant challenged by 0.1 mM DPTA NONOate during 15 min. The symbol (*) and (**) indicate a significant difference compared to the WT strain using *t*-test (P<0.0005 and P<0.0001, respectively).

Transcripts requiring Cwt1p for their proper regulation after exposure to DPTA NONOate were determined using a 1.5 fold cutoff and a statistical-significance analysis with an estimated false-discovery rate of 5% (Table S7). Results showed that Cwt1p is required for proper repression of 77 transcripts including genes involved in iron metabolism (*FTR1*, *PGA10*, *HEM3* and *RBT5*), oxidative stress (*GPX1*, *MDR1*, *SOD3* and *DDR48*) lipid catabolism (*LIP5* and *LIP3*) and the flavohemoglobin *YHB1* (Table 2 and S7). Cwt1p was associated with the promoter of 14 genes among the 77 transcripts requiring this TF for their proper repression (Figure 5).

**Table 2:** Gene expression analysis of Cwt1p target genes in response to nitrosative stress.

Orf19 ID	Gene name	Description	Expression ratio *cwt1* vs wt	Cwt1p occupancy
**Upregulated transcripts**				
**Iron metabolism**				
orf19.7219	*FTR1*	High-affinity iron permease	0.64	1.21
orf19.5674	*PGA10*	Plasma membrane protein involved in heme-iron utilization	0.66	1.12
orf19.1742	*HEM3*	Hydroxymethylbilane synthase; catalyzes conversion of 4-porphobilinogen to hydroxymethylbilane, the third step in the heme biosynthetic pathway;	0.62	1.01
orf19.4215	*FET34*	Protein similar to multicopper ferroxidase	0.66	1.76
orf19.5636	*RBT5*	GPI-anchored cell wall protein involved in hemoglobin utilization	0.48	1.24
**Oxidative stress**				
orf19.87	*GPX1*	Putative thiol peroxidase	0.66	**1.55**
orf19.5604	*MDR1*	Multidrug efflux pump of plasma membrane; member of the MDR family	0.47	**2.85**
orf19.7111.1	*SOD3*	Cytosolic manganese-containing superoxide dismutase, involved in protection against oxidative stress	0.08	1.00
orf19.3461	–	Predicted ORF	0.61	**1.40**
orf19.4082	*DDR48*	Immunogenic stress-associated protein	0.44	1.03
**Lipid metabolism**				
orf19.5179	*LIP5*	Secreted lipase	0.12	1.23
orf19.4856	*LIP3*	Secreted lipase	0.61	1.11
orf19.137	*CST26*	Protein required for incorporation of stearic acid into phosphatidylinositol	0.09	**1.57**
**Nitroastive stress response**				
orf19.3707	*YHB1*	Nitric oxide dioxygenase, acts in nitric oxide scavenging/detoxification	4.73	**11.16**
**Downregulated transcripts**				
**Hexose metabolism**				
orf19.3672	*GAL10*	UDP-glucose 4-epimerase, required for galactose utilization	1.78	1.02
orf19.3651	*PGK1*	Phosphoglycerate kinase; enzyme of glycolysis	1.55	**2.12**
orf19.4618	*FBA1*	Putative fructose-bisphosphate aldolase; enzyme of glycolysis	1.58	**1.55**
orf19.2877	*PDC11*	Protein similar to pyruvate decarboxylase	1.69	**1.54**
orf19.2896	*SOU1*	Enzyme involved in utilization of L-sorbose	1.85	1.07
**Amino acid metabolism**				
orf19.5645	*MET15*	O-acetylhomoserine O-acetylserine sulfhydrylase; involved in sulfur amino acid biosynthesis	1.94	1.20
orf19.5750	*SHM2*	Cytoplasmic serine hydroxymethyltransferase	1.55	1.21
orf19.3554	*AAT1*	Protein described as aspartate aminotransferase	1.62	**3.56**
orf19.946	*MET14*	Putative adenylylsulfate kinase; predicted role in sulfur metabolism	1.59	1.00
orf19.4716	*GDH3*	Protein described as similar to NADP-glutamate dehydrogenase	4.10	1.00
orf19.539	*LAP3*	Protein described as an aminopeptidase	1.53	1.14
orf19.6257	*GLT1*	Putative glutamate synthase	1.84	1.00
orf19.385	*GCV2*	Putative protein of glycine catabolism	1.63	1.01
**Phosphate transport**				
orf19.655	*PHO84*	Protein similar to high-affinity phosphate transporters	1.87	1.07
orf19.2454	*PHO87*	Protein similar to phosphate permeases	1.52	1.00
**Phospholipid metabolism**				
orf19.1063	*FMP44*	*S. cerevisiae* ortholog Gpi18p has dolichyl-phosphate-mannose-glycolipid alpha-mannosyltransferase activity and has role in GPI anchor protein biosynthesis	1.56	1.22
orf19.6396	*NTE1*	Putative patatin-like phospholipase	1.59	1.00
orf19.689	*PLB1*	Phospholipase B	1.52	1.04
orf19.7585	*INO1*	Inositol-1-phosphate synthase; enzyme of inositol biosynthesis	1.55	**1.49**
orf19.6459	*DPP3*	Protein similar to *S. cerevisiae* pyrophosphate phosphatase Dpp1p; required for farnesol biosynthesis	1.67	1.10

Selected GO categories are shown for both down- and upregulated transcripts. Cwt1p binding ratio is also indicated.

Cwt1p was also required for the accurate activation of transcripts related to hexose metabolism (GAL10, PDC11, SOU1, PGK1 and FBA1) and amino acid metabolism (*MET15*, *SHM2*, *AAT1*, *MET14*, *GDH3*, *LAP3*, *GLT1* and *GCV2*). Using the same criteria as described above Cwt1p was found to bind 9 promoter regions among the 94 genes requiring Cwt1p for their proper activation (Figure 5, Table 2 and S7).

## Discussion

### Screening of Transcription Regulatory Mutant Libraries for Potential Modulators of Nitrosative Stress

Although the critical role of NSR in mediating the killing and inhibition of fungal pathogens such as *C. albicans*, *A. fumigatus* and *C. neoformans* [1] has been well established, little is known about the transcriptional regulators that control NO-detoxification process in fungi. In this work we have focused our investigation on the identification of transcriptional regulators controlling nitrosative stress in *C. albicans* using a comprehensive genome-wide screen of mutants for genes encoding TFs as well as components of chromatin remodeling and histone modification complexes. The compiled transcriptional regulatory mutant sets covered approximately 80% (170/213) of the TFs annotated in CGD and 40% (229/500) of proteins annotated to the GO term “regulation of gene expression”. This extensive investigation led to the identification of two transcriptional regulators, Cwt1p and Cta4p acting antagonistically in the control of nitrosative stress tolerance in *C. albicans*. Cta4p is a Zn(II)_2_-Cys_6_ TF that plays a dominant role in nitrosative stress resistance through a direct transcriptional activation the NO-detoxifying enzyme Yhb1p [6]. Cwt1p is also a member of zinc cluster TF family and is a homolog of *S. cerevisiae* Rds2p that plays a key role in regulating genes involved in gluconeogenesis, TCA and glyoxylate cycles [17]. *CWT1* has been a subject of many functional characterizations in *C. albicans*, which showed that this TF is required for cell wall remodeling, morphogenesis and virulence [13], [21]. Up to now, the contribution of Cwt1p, or its homologs Rds2p in the model organism *S. cerevisiae,* to the modulation of nitrosative stress has not been reported. Our work therefore uncovers a new regulator of fungal NSR.

### Expanded Functional Role for the Cwt1p Transcription Factor in Fungi

**Figure 6:**
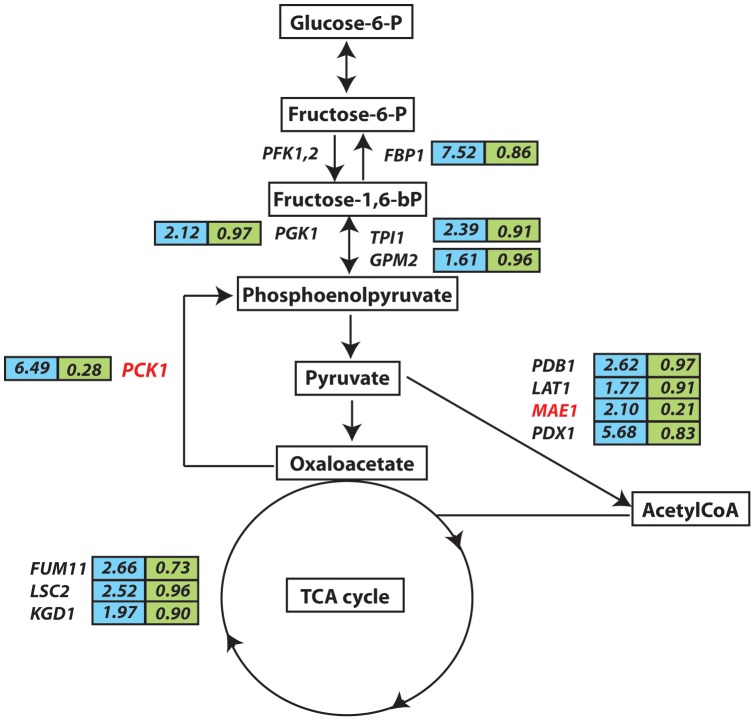
Cwt1p occupancy of gluconeogenesis, pyruvate metabolic and TCA cycle genes. Cwt1p binding ratio and transcription level of genes are shown in blue and green, respectively. Genes bound and requiring Cwt1p for their activation were highlighted in red.

Our genome-wide location profiling demonstrated that Cwt1p occupies the promoters of key gluconeogenesis specific genes, including *PCK1* (Phosphoenolpyruvate carboxykinase) and *FBP1* (Fructose-1,6-bisphosphatase), in addition to many genes implicated in the TCA cycle (*KGD1*, *LSC2*, *FUM11* and *YHM2*) and gluconeogenesis/glycolysis common genes (*PGK1*, *GPM2* and *TPI1*). Expression levels of some of those genes including the key gluconeogenic enzyme, Phosphoenolpyruvate carboxykinase *PCK1* were also downregulated in the *cwt1* strain suggesting that Cwt1p is directly mediating their transcriptional activation (Figure 6 and Table S7). The role of Cwt1p in gene expression regulation of carbohydrate metabolism thus appears to be conserved between *C. albicans* and *S. cerevisiae* [17]. However, Cwt1p was found to be associated with the promoters of genes related to pyruvate metabolism (*PDB1*, *LAT1*, *MAE1* and *PDX1*) specifically in *C. albicans*. The list of Cwt1p promoter targets was also enriched for genes from a variety of GO categories that has not previously been related to Rds2p, including translation, proteolysis, vesicle-mediated transport and amino acid metabolism (Table S3). Another target of Cwt1p in *C. albicans* includes *YHB1*, encoding the key NO-detoxifying enzyme flavohemoglobin/nitric oxide dioxygenase. Our investigations demonstrated that Cwt1p is a negative modulator of nitrosative stress resistance through a direct transcriptional control of *YHB1*. The role of Cwt1p in mediating NSR is thus novel and unique to *C. albicans* since *S. cerevisiae rds2* mutant didn’t show any growth alteration in the presence of different DPTA NONOate concentrations (data not shown).

### Transcriptional Regulatory Network Mediating Nitrosative Stress Response in *C. albicans*

The transcriptional response to nitrosative stress has been well elucidated in several fungi including *C. albicans* [7]. Generally, in response to DPTA NONOate *Candida* cells upregulates genes involved in the oxidative stress response, iron uptake and sulfur assimilation. In our investigation we reexamined the *C. albicans* nitrosative stress transcriptome using GSEA. This method ranks genes according to their changes in transcript abundance and compares them to a large collection (5689 gene sets) of expression profiling, ChIP-chip, and GO categories data in addition to different *S. cerevisiae* synthetic genetic interactions and protein-protein interaction datasets. This analysis revealed an interesting and unexpected correlation with the ciclopirox olamine expression profiling data (Table 1 and Figure S1). Ciclopirox olamine is an anti-infective agent that seems to act as an iron chelator [22]. Similarly, as reported in this work for the DPTA NONOate, ciclopirox olamine induces the transcription of many genes involved in iron uptake and oxidative stress [23] suggesting that this compound might cause nitrosative stress. This hypothesis is buttressed by the fact that, like the DPTA NONOate, ciclopirox olamine has an ethanolamine chemical group that is a potential nitric oxide donor. Additional support of this assumption came from recent work undertaken in rats which demonstrated that perfusion with ciclopirox significantly increased the endogenous level of NO [24].

Intriguingly, nitrosative stress responsive genes exhibit a convincing correlation with the transcriptional programs of *C. albicans* interacting with host-cells, such as polymorphonuclear neutrophils [14]. This resemblance strongly supports the hypothesis that immune cells, such as neutrophils, inhibit *C. albicans* growth through the release of nitric oxide radicals. Taking into consideration the resistance of *cwt1* to NO radicals, this mutant should have a beneficial capacity to circumvent the immune system defense and an *in vivo* fitness gain. However, previous works undertaken in mouse model have shown that the virulence of *cwt1* mutant was similar to its parental background strain [13], suggesting a minor contribution of NO-resistance to *Candida* pathogenicity in this systemic infection model.

In conclusion, we have characterized a new transcriptional regulator of NSR, which act as a cis-repressor of the key NO-detoxifying enzyme Yhb1p as well as other NO-responsive transcripts. Cwt1p is also required a proper activation of many NO-responsive genes which are its direct targets implying that this TF, as reported for its homolog Rds2p in *S. cerevisiae* [17], behaves both as a repressor and activator. Cwt1p was also associated with the promoter of transcriptional regulators including different TFs (Zcf18p, Sut2p, Cas1p, Lys14p) related to different biological functions and two transcriptional repressors Mig1p and Nrg1p controlling genes involved in carbohydrate metabolism. This suggests that Cwt1p is a high-level hierarchical regulator controlling different biological processes in *C. albicans*.

## Materials and Methods

### *C. albicans* Strain Construction, Growth Media and Conditions

For general propagation and maintenance conditions, the strains were cultured at 30°C in yeast-peptone-dextrose (YPD) medium supplemented with uridine (2% Bacto peptone, 1% yeast extract, 2% dextrose, and 50 µg/ml uridine). For gene expression profiling of cells treated with Dipropylenetriamine NONOate (DPTA NONOate; Cayman chemical), saturated overnight cultures of the wild type SN152 (*arg4Δ/arg4Δ, leu2Δ/*LEU2, *his1Δ/*HIS1, URA3/*ura3Δ,* IRO1/*iro1Δ*) and *cwt1* mutant (Homann TF collection [8]) were diluted to a starting optical density at 600 nm (OD_600_) of 0.1 in 100 ml fresh YPD buffered with 50 mM sodium phosphate pH 7.3, grown at 30°C to an OD_600_ of 0.8 and then split into two 50 ml cultures. DPTA NONOate was added to the experimental culture to a final concentration of 0.1 mM. An equal volume of YPD buffered with 50 mM sodium phosphate pH 7.3 was added to the control culture. For the profiling experiment using microarray, the cultures were incubated for 15 min. Different time-points (5, 15, 60 and 180 min) were considered for the qPCR experiment assessing the transcript level of *YHB1*. Cells were harvested by centrifugation and stored at −80°C.

For reintegration experiments, the *CWT1* gene was reintegrated into the null mutant *cwt1* strain from the Homann TF deletion library [8]. The wild-type *CWT1* gene was amplified from genomic DNA using oligonucleotides Ctw1RevF1and Ctw1RevR1 (Table S8) and Expand high fidelity polymerase (Roche). The PCR fragment was digested with restriction enzymes *SalI* and *MluI* and cloned in the same sites of the CIp30 vector [25]. The plasmid was sequenced to confirm the integrity of the *CWT1* gene. Plasmid CIp30-*CWT1* was digested with the *StuI* restriction enzyme and used to transform the *cwt1* mutant strain. The arginine-positive colonies were analyzed by PCR and the obtained wild-type fragment confirmed the reintegration of the *CWT1* gene.

### Growth Inhibition Assays

NO inhibition assay was performed as described by [6]. Library screening was performed at two DPTA NONOate concentrations: 0.3 mM and 1 mM. Growth inhibition rate was normalized relative to the 0 mM DPTA NONOate condition and was compared to their respective parental strain (SN152 for Homann’s deletion library [8], BWP17 for Sanglard’s insertion library, DAY185 for Mitchell’s insertion library [12] and SN250 for Noble’s deletion library [9]). The growth defect of the *cwt1* and *cta4* 3 was confirmed by an independent NO inhibition assay using different DPTA NONOate concentrations: 0, 0.1, 0.2, 0.3, 0.4, 0.5, and 1 mM.

### Quantitative Real Time PCR

Cells for gene expression analysis by qPCR were prepared as described for the microarray experiment except the fact that cultures were incubated with DPTA NONOate at different time-points (5, 15, 60 and 180 min). cDNA was synthesized from 2 µg of total RNA using the reverse-transcription system (50 mM Tris-HCl, 75 mM KCl, 5 mM DTT, 3 mM MgCl2, 400 nM oligo(dT)15, 20 ng random octamers, 0.5 mM dNTPs, 200 units Superscript III reverse transcriptase; Invitrogen). The mixture was incubated for 30 min at 50°C. cDNAs were then treated with 2U of RNase H (Promega) for 20 min at 37°C followed by heat inactivation of the enzyme at 80°C for 10 min. Aliquots were used for qPCR, which was performed using the Mx3000P QPCR System (Agilent) with the QuantiTect SYBR Green PCR master mix (Qiagen). Cycling was 10 min at 95°C followed by 40 cycles (95°C, 10 s; 58°C, 15 s; 72°C, 15 s). Samples were done in triplicate and means were used for calculations. Fold changes were estimated by using the coding sequence of the *C. albicans* ACT1 ORF as a reference. Fold enrichments of the tested coding sequences were estimated using the comparative ΔΔCt method [26]. Primer sequences used for this analysis are summarized in Table S8.

### ChIP-CHIP and ChIP-qPCR

*CWT1* was TAP-tagged *in vivo* with a TAP-*URA3* PCR product in BWP17 strain as described by [27]. Transformants were selected on YPD –ura plates and correct integration of the TAP-tag was checked by PCR and sequencing. CWT1-TAP expressed in BWP17 strain was fully functional based on complementation of the nitrosative stress sensitivity phenotype. Deleting the non-tagged allele in the CWT1-TAP strain revealed that this strain has a comparable sensitivity to the single knockout strain and even to the parental strain. ChIP-chip was performed as previously described [27], [28] with few modifications. Briefly, cells were grown to an optical density at 600 nm of 1.8 in 45 ml of YPD. Tiling arrays were co-hybridized with tagged immunoprecipitated (Cy5-labeled) and mock immunoprecipitated (untagged BWP17 strain; Cy3-labeled) DNA samples. Binding locations were determined in duplicate ChIP-chip experiments using microarray containing 5449 70-mer oligonucleotides representative of all intergenic regions. Data handling and analysis were carried out using Genespring v.7.3 (Agilent Technologies, Palo Alto, CA). Statistical analysis using the Welch t test with a false-discovery rate of 5% and 1.6 fold enrichment cutoff was used. For qPCR validations, *in vivo* TAP-tagged Cwt1p was ChIPed as described for chip-chip. PCR reactions were performed exactly as for gene expression analysis. Fold enrichments of tested promoter sequences were estimated by using the coding sequence of the *C. albicans ACT1* ORF as a reference. Oligonucleotide sequences are given in Table S8.

ChIP-qPCR was performed using 1 ng of TAP-ChIPed DNA or total genomic DNA extracted from the whole cell extract (WCE). Cycling was for 15 min at 95°C, followed by 45 cycles of 95°C for 10 s, 58°C for 15 s, and 72°C for 15 s. All samples were tested in duplicates and means were used for further calculations.

### RNA Preparation

Total RNA was extracted as described in [29] with few modifications. Briefly, samples stored at −80°C were placed on ice and RNeasy buffer RLT was added to pellets at a ratio of 1∶1 [vol/vol] buffer/pellet. The suspended pellet was placed back on ice and divided into 1 ml aliquots in 2 ml screw cap microcentrifuge tubes containing 0.6 ml of 3 mm diameter acid-washed glass beads. Samples were homogenized 5 times, 1 min each in a FastPrep®-24 bead beater for 60 s at 6 m/s. After the homogenization the Qiagen RNeasy protocol was followed as recommended. Total RNA samples were eluted in RNAse free H_2_O. RNA quality and integrity were assessed using an Agilent 2100 bioanalyzer.

### Microarray Hybridization and Processing

cDNA labeling and microarray production were performed as described by Nantel et al. [30]. Briefly, 14 µg of total RNA was reverse transcribed using 9 ng of oligo(dT)_21_ in the presence of Cy3 or Cy5-dCTP (Invitrogen) and 400 U of Superscript III reverse transcriptase (Invitrogen) in a 40 µl reaction. After cDNA synthesis, template RNA was degraded by adding 2.5 units RNase H (Promega) and 1 µg RNase A (Pharmacia) followed by incubation for 15 min at 37°C. The labeled cDNAs were purified with QIAquick PCR Purification Kit (Qiagen). DNA microarrays were processed and analyzed as above for ChIP-chip experiment. NO-responsive transcripts and genes requiring Cwt1p for their proper regulation were determined using the Welch *t*-test with a false-discovery rate of 5% and 1.5 fold enrichment cutoff.

### Gene Set Enrichment Analysis (GSEA)

Gene Set Enrichment Analysis (GSEA), was used to determine whether defined lists (or sets) of genes exhibit a statistically significant bias in their distribution within a ranked gene list (see http://www.broadinstitute.org/gsea/for details [19]. This required initially the construction of an extensive gene set and annotation database using publicly available data from CGD, SGD and BioGRID, together with transcription factor binding data from all currently published ChIP-chip experiments, our own TF motif database, lists of modulated genes from both transcriptional profiling experiments, and genetic-association data obtained from SGA screens measuring cell growth.

### MEME Analysis

For de novo identification of the consensus-binding site, the sequences were analyzed with the Multiple EM for Motif Elucidation (MEME) program (http://meme.sdsc.edu/meme/cgi-bin/meme.cgi). Sequences of 300 bp upstream the ATG of 17 Cwt1p targets (using a 3 fold binding enrichment cutoff) were extracted and used for min-motif detection. The motifs were allowed to have any sequence and length (between 6 and 20 bp) and could be present anywhere in the sequence.

## Supporting Information

Figure S1 (TIF)

Table S1 Results of the primary screen of *C. albicans* mutant collections using 0.3 mM and 1 mM of DPTA NONOate.
(XLS)

Table S2 ChIP-chip datasets showing DNA loci occupied by Cwt1p.
(XLSX)

Table S3 Gene Ontology analysis of Cwt1p-targets.
(XLSX)

Table S4 The five most significant motifs in the core promoter sequences from −300 bp to 0 bp relative to the ATG codon, of 17 Cwt1p-bound promoter regions (top ranked Cwt1p targets) as identified by the MEME algorithm.
(DOCX)

Table S5 NO-responsive genes identified by analyzing the transcriptional profile of wt *C. albicans* exposed to DPTA NONOate for 15 min.
(XLSX)

Table S6 GSEA analysis of DPTA NONOate-differentially expressed in the wt strain.
(XLSX)

Table S7 Genes differentially expressed in *cwt1* mutant after treatment with 0.1 mM DPTA NONOate during 15 min.
(XLSX)

Table S8 List of primers used in this study.
(DOCX)
