# Supplementary figures and images for: A Novel Role for the Transcription Factor Cwt1p as a Negative Regulator of Nitrosative Stress in Candida albicans
Source: PLoS One. 2012 Aug 29;7(8):e43956. doi: 10.1371/journal.pone.0043956 (PMC3430608; doi:10.1371/journal.pone.0043956)

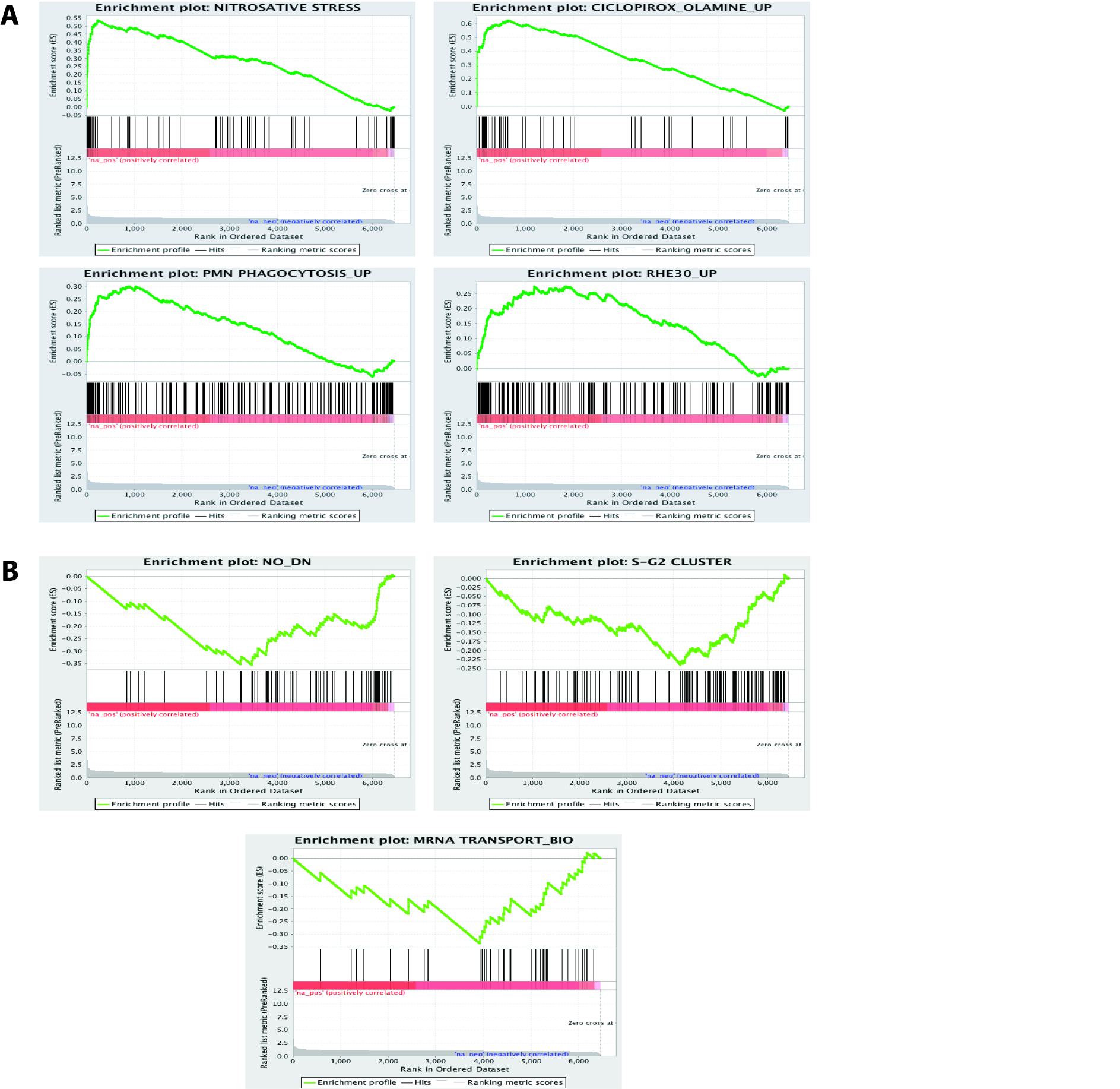

Supplement: Figure S1 — (TIF) [file pone.0043956.s001.tif]
