# Supplementary material for: A Novel Role for the Transcription Factor Cwt1p as a Negative Regulator of Nitrosative Stress in Candida albicans
Source: PLoS One. 2012 Aug 29;7(8):e43956. doi: 10.1371/journal.pone.0043956 (PMC3430608; doi:10.1371/journal.pone.0043956)
Supplement: Table S4 — The five most significant motifs in the core promoter sequences from −300 bp to 0 bp relative to the ATG codon, of 17 Cwt1p-bound promoter regions (top ranked Cwt1p targets) as identified by the MEME algorithm. (DOCX) [file pone.0043956.s005.docx]

| **Motif** | **Sequence logo** | **Consensus** | **Number** | **E-value** |
| --- | --- | --- | --- | --- |
| **1** | 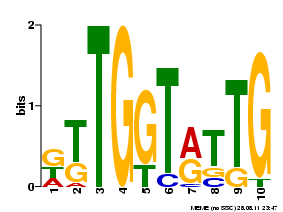 | [GTA][TG]TG[GT]T[AG]TTG | 17 | 7.0e-000 |
| **2** | 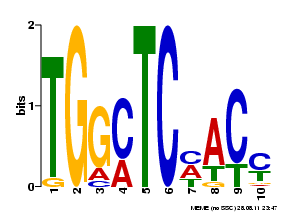 | TGG[CA]TC[ACT][AT][CT][CT] | 13 | 1.1e-003 |
| **3** | 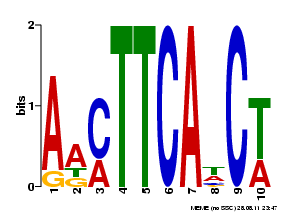 | A[AGT][CA]TTCA[TA]C[TA] | 13 | 2.7e-003 |
| **4** | 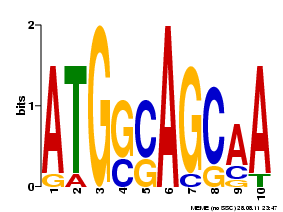 | ATG[GC][CG]AG[CG][AC]A | 9 | 4.1e-003 |
| **5** | 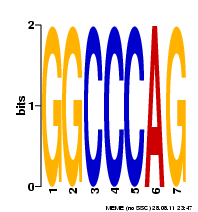 | GGCCCAG | 2 | 5.4e-004 |

**Table S4.** The five most significant motifs in the core promoter sequences from -300 bp to 0 bp relative to the ATG codon, of 17 Cwt1p-bound promoter regions (top ranked Cwt1p targets) as identified by the MEME algorithm.
