# Supplementary material for: A Novel Role for the Transcription Factor Cwt1p as a Negative Regulator of Nitrosative Stress in Candida albicans
Source: PLoS One. 2012 Aug 29;7(8):e43956. doi: 10.1371/journal.pone.0043956 (PMC3430608; doi:10.1371/journal.pone.0043956)
Supplement: Table S8 — List of primers used in this study. (DOCX) [file pone.0043956.s009.docx]

**Table S7**. Primers used in the study

| **Primer name** | **Primer sequence** | **Description** | **Purpose** |
| --- | --- | --- | --- |
| CWT1 F1 | atggtattaaacgaaaaagttgctgtttcagctttactataaggagagatagatacaatattcccagttgtatagtgggtaattttatccccattgatcctGGTCGACGGATCCCCGGGTT | 5' CWT1 PCR cassette | C-terminal TAP-tagging |
| CWT1 R1 | agaagaggatgagcaagaagatgaagatattgtaatggaggaggaagatgatgagtagtttagatttaacaaataatttacagttgtgttaatacacgataTCGATGAATTCGAGCTCGTT | 3' CWT1 PCR cassette | C-terminal TAP-tagging |
| CWT1 ExF1 | gcgggaatagcacgactaac | CWT1 , forward-external | Validation of 5’ cassette insertion |
| CWT1 ExR1 | tcgaacgcaaaagagggata | CWT1 , reverse-external | Validation of 3’ cassette insertion |
| U1 | TTGAAGGATTAAAACAGGGAGC | URA3, forward | Validation of 3’ cassette insertion |
| U2 | ATACCTTTTACCTTCAATATCTGG | URA3, reverse | Validation of 5’ cassette insertion |
| YHB1 F1  YHB1 R1  HGT7 F1 | Tttccgcggttttgtgtaat  Tgctcaattgttcagtcaagttt  Ccttcaagaattgttctgaaatc | YHB1, forward  YHB1, reverse  HGT7, forward | QPCR-ChIP validation |
| HGT7 R1  FBP1 F1  FBP1 R1 | ttctgggtcactcactaaaacg  Aactgggccactcattatgc  tttccttttatccgccattc | HGT7, reverse  FBP1, forward  FBP1, reverse | QPCR-ChIP validation |
| CDR1 F1  CDR1 R1  MDR1 F1 | Ttttcgggaaagtttcaatca  acacagccgccctcactc  Agattgatgcgaatgggaac | CDR1, forward  CDR1, reverse  MDR1, forward | QPCR-ChIP validation |
| MDR1 R1  PGK1 F1  PGK1 R1 | aaaaaggcggatttactcctg  Caattgaatgaaatgaggagtga  ctctcttccagttgtgcgtct | MDR1, reverse  PGK1, forward  PGK1, reverse | QPCR-ChIP validation |
| MIG1 F1  MIG1 R1  NRG1 F1 | Ccaatctctttctccgtggt  gaaaaagaaacgacgccaac  Gatgaaagcaaggaggggtta | MIG1, forward  MIG1, reverse  NRG1, forward | QPCR-ChIP validation |
| NRG1 R1 QDR1 F1  QDR1 R1  Yhb1q F1  Yhb1q R1  ACT1 qF1  ACT1 qR1  Cwt1RevF1  Cwt1RevR1 | gagcgagttcgttttggatt  Cgacgacagatctcaggattt  gcctaaagaaaaggaaataaaatagc  TGAAAGTTGCTCCTCCTGCT  TCTTTGAAAAGGTTGCCGA  gaagcccaatccaaaaga  cttctggagcaactctcaattc  Cgcgtcgactatgattggtgaatcaatgttgttgtg  cgacgcgtctaaggatcaatggggataaaat | NRG1, reverse  QDR1, forward  QDR1, reverse  YHB1, forward  YHB1, reverse  ACT1, forward  ACT1, reverse  CWT1, forward  CWT1, reverse | QPCR-ChIP validation  _­_  QPCR-ChIP validation  QPCR expression quantification  QPCR expression quantification  Revertant construction |
| Yhb1_F | TTGATAAATGGTGATGTTTAGTGG | YHB1 promoter, (-3924 to -4523), Forward | Tiled ChIP-qPCR |
| Yhb1_R | AAAAGGATAGTTGATTCCTCTTGG | YHB1 promoter, (-3924 to -4523), Reverse |  |
| Yhb2_F | CTAGCTTATTGTTCTTTTTCATTCTCC | YHB1 promoter, (-3684 to -3923), Forward |  |
| Yhb2_R | TGCATTTGAATAAACGGCATC | YHB1 promoter, (-3684 to -3923), Reverse |  |
| Yhb3_F | TTACCCGTTTCACTTTCTGC | YHB1 promoter, (-3444 to -3683), Forward |  |
| Yhb3_R | CACGCGTGCGTTTTAATTC | YHB1 promoter, (-3444 to -3683), Reverse |  |
| Yhb4_F | AAAGCAAAGCAAAGCAAAGC | YHB1 promoter, (-3203 to -3443), Forward |  |
| Yhb4_R | GTTGCCTGTCAACAACAAGC | YHB1 promoter, (-3203 to -3433), Reverse |  |
| Yhb5_F | AACTTGTGTTTGTTGACGTTTCA | YHB1 promoter, (-2964 to -3203), Forward |  |
| Yhb5_R | TCTTTCCCCTTGGAACTTTG | YHB1 promoter, (-2964 to -3203), Reverse |  |
| Yhb6_F | TGAAAGATCTATTGTTCTTCTTCTTTT | YHB1 promoter, (-2724 to -2963), Forward |  |
| Yhb6_R | GAGGCACCATCACTATTACTAAACA | YHB1 promoter, (-2724 to -2963), Reverse |  |
| Yhb7_F | TTTCAATCCTTTCCCCTTTTC | YHB1 promoter, (-2484 to -2723), Forward |  |
| Yhb7_R | CAAATCTTTTCTTCTTGGTGACG | YHB1 promoter, (-2484 to -2723), Reverse |  |
| Yhb8_F | CAATCAATCAATGGAAATTAACGA | YHB1 promoter, (-2244 to -2483), Forward |  |
| Yhb8_R | AATGCAGAAAAGCGCAAAAT | YHB1 promoter, (-2244 to -2483), Reverse |  |
| Yhb9_F | GCCACAACAACAACAGAAACA | YHB1 promoter, (-2004 to -2243), Forward |  |
| Yhb9_R | ATTTATGGGCCGATCTGAAA | YHB1 promoter, (-2004 to -2243), Reverse |  |
| Yhb10_F | TTTCCACTTGTTAACCAACCAA | YHB1 promoter, (-1764 to -2003), Forward |  |
| Yhb10_R | CGACAGATAACGTAAAAATGTGGA | YHB1 promoter, (-1764 to -2003), Reverse |  |
| Yhb11_F | TTGTCCAAAGGTGTTCCACA | YHB1 promoter, (-1764 to -2003), Forward |  |
| Yhb11_R | TGGCATATGTTGTTGTGGTTG | YHB1 promoter, (-1764 to -2003), Reverse |  |
| Yhb12_F | TCTAGAAAATATGGTTACGGCTTT | YHB1 promoter, (-1524 to -1763), Forward |  |
| Yhb12_R | CATCTAATTTCTTCATACGGTCGTT | YHB1 promoter, (-1524 to -1763), Reverse |  |
| Yhb13_F | TTCATTAAGCCAACATTGAGAAAA | YHB1 promoter, (-1284 to -1523), Forward |  |
| Yhb13_R | TGCAAATTTACGTTAAGCTTCG | YHB1 promoter, (-1284 to -1523), Reverse |  |
| Yhb14_F | CAATTCTTCCCCTCCGAGTT | YHB1 promoter, (-1284 to -1523), Forward |  |
| Yhb14_R | TGTTGCTGCTACACCAAGAAG | YHB1 promoter, (-1284 to -1523), Reverse |  |
| Yhb15_F | CTTTATTCCCGCGATTTGC | YHB1 promoter, (-1044 to -1283), Forward |  |
| Yhb15_R | CATTCATTGATTGATTGATTGTCTT | YHB1 promoter, (-1044 to -1283), Reverse |  |
| Yhb16_F | TCCTCCCCTGATTTGATCTG | YHB1 promoter, (-804 to -1043), Forward |  |
| Yhb16_R | TTGCTCTATAGGGCGTTGCT | YHB1 promoter, (-804 to -1043), Reverse |  |
| Yhb17_F | GAAGACGCCTCAAACGAAAA | YHB1 promoter, (-564 to -803), Forward |  |
| Yhb17_R | GATGTTGTCGGTTGTACGAAA | YHB1 promoter, (-564 to -803), Reverse |  |
| Yhb18_F | TACCAACTACACTGCTCAATTGTT | YHB1 promoter, (-444 to -563), Forward |  |
| Yhb18_R | AACTTGTCTCATTCTCTCGGTCTT | YHB1 promoter, (-444 to -563), Reverse |  |
| Yhb19_F | TCCCAACTCCTCCATCATTC | YHB1 promoter, (-264 to -443), Forward |  |
| Yhb19_R | TTTCCGCGGTTTTGTGTAAT | YHB1 promoter, (-264 to -443), Reverse |  |
| Yhb20_F | TATTACCGTCGGGGAGCAT | YHB1 promoter, (-144 to -263), Forward |  |
| Yhb20_R | GGAAAACAAACGTACATGAAAAA | YHB1 promoter, (-144 to -263), Reverse |  |
| Yhb21_F | TTACTTCCCCATGATTTCCA | YHB1 promoter, (-1 to -143), Forward |  |
| Yhb21_R | GCTGCTCTGTTCTAAAGTTGTCT | YHB1 promoter, (-1 to -143), Reverse |  |
